# Examining the U-shaped relationship of sleep duration and systolic blood pressure with risk of cardiovascular events using a novel recursive gradient scanning model

**DOI:** 10.3389/fcvm.2023.1210171

**Published:** 2023-09-14

**Authors:** Shuo Yang, Nanxiang Zhang, Zichao Liang, Yuduan Han, Hao Luo, Yingfeng Ge, Jianan Yin, Chonglong Ding, Chao Li, Qitong Zhang, Jinxin Zhang

**Affiliations:** ^1^ Department of Medical Statistics, School of Public Health, Sun Yat-sen University, Guangzhou, China; ^2^ Department of Clinical Medicine, Zhongshan School of Medicine, Sun Yat-sen University, Guangzhou, China

**Keywords:** sleep duration, systolic blood pressure, cardiovascular diseases, U-shape, retrospective study

## Abstract

**Background:**

Observational studies have suggested U-shaped relationships between sleep duration and systolic blood pressure (SBP) with risks of many cardiovascular diseases (CVDs), but the cut-points that separate high-risk and low-risk groups have not been confirmed. We aimed to examine the U-shaped relationships between sleep duration, SBP, and risks of CVDs and confirm the optimal cut-points for sleep duration and SBP.

**Methods:**

A retrospective analysis was conducted on NHANES 2007–2016 data, which included a nationally representative sample of participants. The maximum equal-odds ratio (OR) method was implemented to obtain optimal cut-points for each continuous independent variable. Then, a novel “recursive gradient scanning method” was introduced for discretizing multiple non-monotonic U-shaped independent variables. Finally, a multivariable logistic regression model was constructed to predict critical risk factors associated with CVDs after adjusting for potential confounders.

**Results:**

A total of 26,691 participants (48.66% were male) were eligible for the current study with an average age of 49.43 ± 17.69 years. After adjusting for covariates, compared with an intermediate range of sleep duration (6.5–8.0 h per day) and SBP (95–120 mmHg), upper or lower values were associated with a higher risk of CVDs [adjusted OR (95% confidence interval) was 1.20 (1.04–1.40) for sleep duration and 1.17 (1.01–1.36) for SBP].

**Conclusions:**

This study indicates U-shaped relationships between SBP, sleep duration, and risks of CVDs. Both short and long duration of sleep/higher and lower BP are predictors of cardiovascular outcomes. Estimated total sleep duration of 6.5–8.0 h per day/SBP of 95–120 mmHg is associated with lower risk of CVDs.

## 1. Introduction

Cardiovascular diseases (CVDs) continue to be the foremost cause of both morbidity and mortality on a global scale (1). Recent studies have suggested that there may be U-shaped associations between systolic blood pressure (SBP), sleep duration, and CVDs (2–8). Thus, determining the optimal range of SBP and sleep duration is crucial for reducing the risk of CVDs.

When examining the relationships between continuous explanatory variables and health-related outcomes in medical research, it is commonly recommended to investigate U-shaped relationships when non-linear effects are suspected (9–11). If we make the simplifying assumption that these continuous variables exhibit a linear correlation with prognosis and directly incorporate them into the construction of regression models, this approach will lead to a significant increase in the residuals of regression analysis. On the other hand, the epidemiologists consequently may fail to find a fundamental clue to formulate intervention measures. Although Cox regression models supplemented by flexible smoothing techniques (12–14), such as penalized splines and restricted cubic splines, can handle the U-shaped effects of continuous variables, many clinical and epidemiological researchers prefer to categorize continuous explanatory variables into high-risk and low-risk groups (15, 16). Optimal cut-points can identify crucial predictor thresholds, facilitate the development of patient classification schemes, and assist in clinical treatment strategies. However, determining appropriate cut-points becomes critical when clinical reference ranges are unavailable or cannot be directly applied to populations with distinct characteristics (17–21).

Two methods are utilized to discretize continuous independent variables in biostatistical analysis. One of them is the data-oriented cut-points approach (22, 23), which involves utilizing percentiles like median or quartiles based on the distribution of continuous variables. Although this method is easy to implement, it can produce arbitrary cut-points that do not consider the relationship with survival outcomes and may lead to inaccurate estimates of the actual effects (24). The other approach is the maximum statistic or minimum *p*-value approach (25), which chooses a cut-point with maximum *χ*^2^ statistic as the optimal cut-point for binary outcomes. However, the above two discretization approaches have a high probability of dividing individuals with similar risk into different groups, leading to inconsistent discretization results for high- and low-risk groups.

To address the limitations of conventional discretization methods and fulfill the requirement of identifying optimal cut-points for continuous predictors that exhibit a U-shaped relationship with outcomes, our team proposed two novel methods to discretize the single non-monotonic continuous variable, namely, “two cut-points with maximum odds ratio (OR) value method” (26) and “optimal equal-hazard ratio with minimum Akaike information criterion (AIC) value method” (27), which have been widely validated by peer review consensus. The OR or RR values obtained by our original methods not only directly respond to clinical needs but also optimize the evaluation from a statistical methodology perspective. Due to the universality of our proposed methods, the methods were quickly applied by domestic and foreign scholars to solve their practical problems, such as optimal cut-points identification of biomarkers (i.e., red blood cell distribution width for prognostic significance, serum creatinine for kidney injury after lung transplantation, and hemoglobin for surgical coronary revascularization). However, extending this discretization method to multiple non-monotonic independent variables can be challenging, as it requires a more complex consideration on either theoretical assumption or algorithm implementation. Currently, our team has developed a novel “recursive gradient scanning method” for the discretization of multiple non-monotonic independent variables simultaneously. This approach enables us to prioritize critical intervention measures and achieve targeted goals efficiently. The proposed method will provide a theoretical basis and algorithmic support for identifying significant influencing factors and constructing intervention programs.

This study utilized data from the National Health and Nutrition Examination Surveys (NHANES) covering 12 years to examine the relationships between sleep duration, SBP, and risks of CVDs. In addition, this study aimed to identify the optimal cut-points for sleep duration and SBP with CVDs as the outcome of interest, test the new method on real-world data, and compare its performance with other existing methods for discretizing multiple non-monotonic independent variables.

## 2. Materials and methods

### 2.1. Sample and design

The NHANES surveys use a complex, multistage, probability sampling design to create a representative sample of the civilian, non-institutionalized US population, and are conducted in a series of cross-sectional population-based surveys. Each year, about 5,000 individuals are examined, and data are released to the public in 2-year cycles. NHANES datasets have detailed information on data collection procedures and analytic guidelines provided elsewhere (28, 29). To gather information on CVDs, questionnaires were added to the surveys from 2007 to 2016, and this study used a total of five cycles (NHANES 2007–2016).

### 2.2. Definition of outcome

The presence of CVDs was ascertained using a combination of self-reported physician diagnoses and standardized medical status questionnaires, which were completed during individual interviews. The participants were specifically asked if a healthcare professional had ever informed them of having congestive heart failure (CHF), coronary heart disease (CHD), angina pectoris, heart attack, or stroke. Those who answered “yes” to any of the above were considered as having CVDs, and the outcome was converted to a dichotomous variable. Participants who responded with “did not know” were excluded from the analysis.

### 2.3. Sleep duration and SBP assessment (ascertainment of exposure)

NHANES datasets use responses to the question “How much sleep do you usually get at night on weekdays or workdays?” to obtain information on sleep duration. In cases where individuals reported a sleep duration of ≥12 h, this value was coded as 12. To minimize the risk of inaccurate sleep duration data and the potential impact of poor health on the study results, we opted to exclude individuals with missing sleep duration and those who reported sleeping less than 4 h. An average of three consecutive blood pressure measurements taken after resting quietly for 5 min was used to determine the SBP and diastolic blood pressure (DBP).

### 2.4. Covariate data collection

In our analysis, sociodemographic and lifestyle characteristics were considered covariates based on previously published studies (30–32). The study took multiple sociodemographic factors into account, such as gender, age, race (Mexican American, Other Hispanic, Non-Hispanic White, Non-Hispanic Black, and Other Race), poverty income ratio (PIR) (≤130%, 131%–185%, and ≥186%) (33), marital status (unmarried, married or living with partners, divorced or separated), and educational level (≤9th grade, 9–11th grade, high school grade, college and above). In this study, lifestyle characteristics were defined as physical activity, calculated in metabolic equivalents (METs) minutes per week and classified as <600 MET-min/week or ≥600 MET-min/week (34), smoking status (never, ever, and current), and alcohol consumption (never, ever, moderate, and excessive intake).

### 2.5. Statistical analysis

All statistical analyses in this study were adjusted for the complex sampling design of NHANES.

#### 2.5.1. Descriptive analysis and modeling

Participant characteristics were summarized using weighted means and standard deviations for continuous variables and weighted counts and percentages for categorical variables. Differences between participants with and without CVDs were assessed using Rao–Scott *χ*^2^ for categorical variables and independent *t*-tests for continuous variables. A multivariable logistic regression model was used to examine the association between sleep duration, SBP, and the risk of CVDs, with the lower risk group serving as the reference category.

#### 2.5.2. Graphical diagnostic plot

The semiparametric models with penalized B-splines (P-splines) were fitted using the R package “SemiPar” (35). This approach balances the goodness of fit and variance to curve the relationship and assess the statistical significance of the non-linear term.

#### 2.5.3. Find two optimal cut-points for each continuous explanatory variable as original cut-points

If the visual representation of the curve indicates a U-shaped relationship (*df* > 2 using semiparametric regression analysis), then the “two cut-points with maximum OR value method (26)” was used to identify the original upper and lower cut-points of the continuous explanatory variable at which the OR reaches its maximum.

#### 2.5.4. The recursive gradient scanning method for the discretization of multiple non-monotonic independent variables

**Figure 1:**
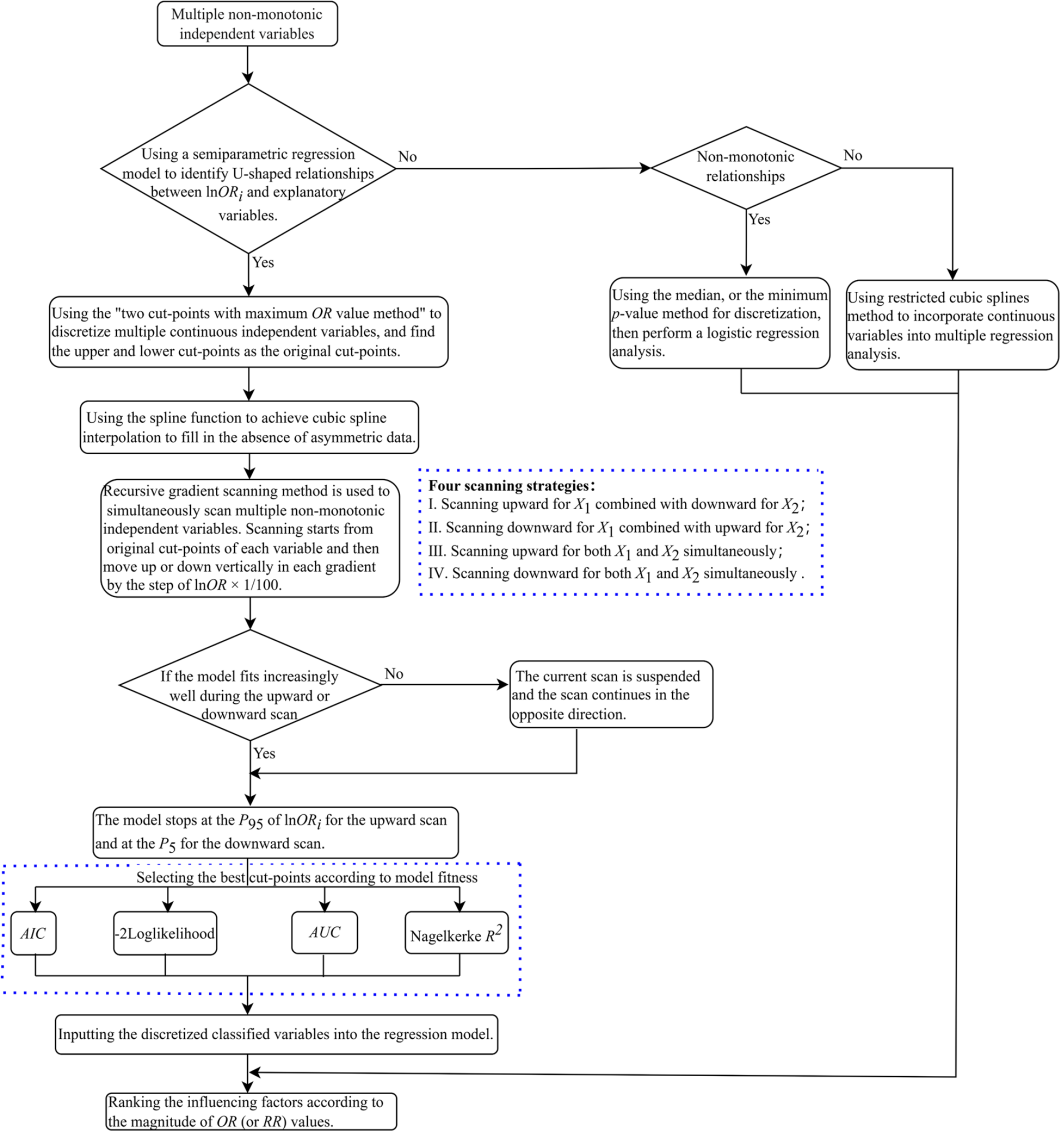
The process of calculation implementation.

The details of the methods raised by us to discretize multiple non-monotonic independent variables simultaneously are described as follows (depicted in Figure 1).

**Figure 2:**
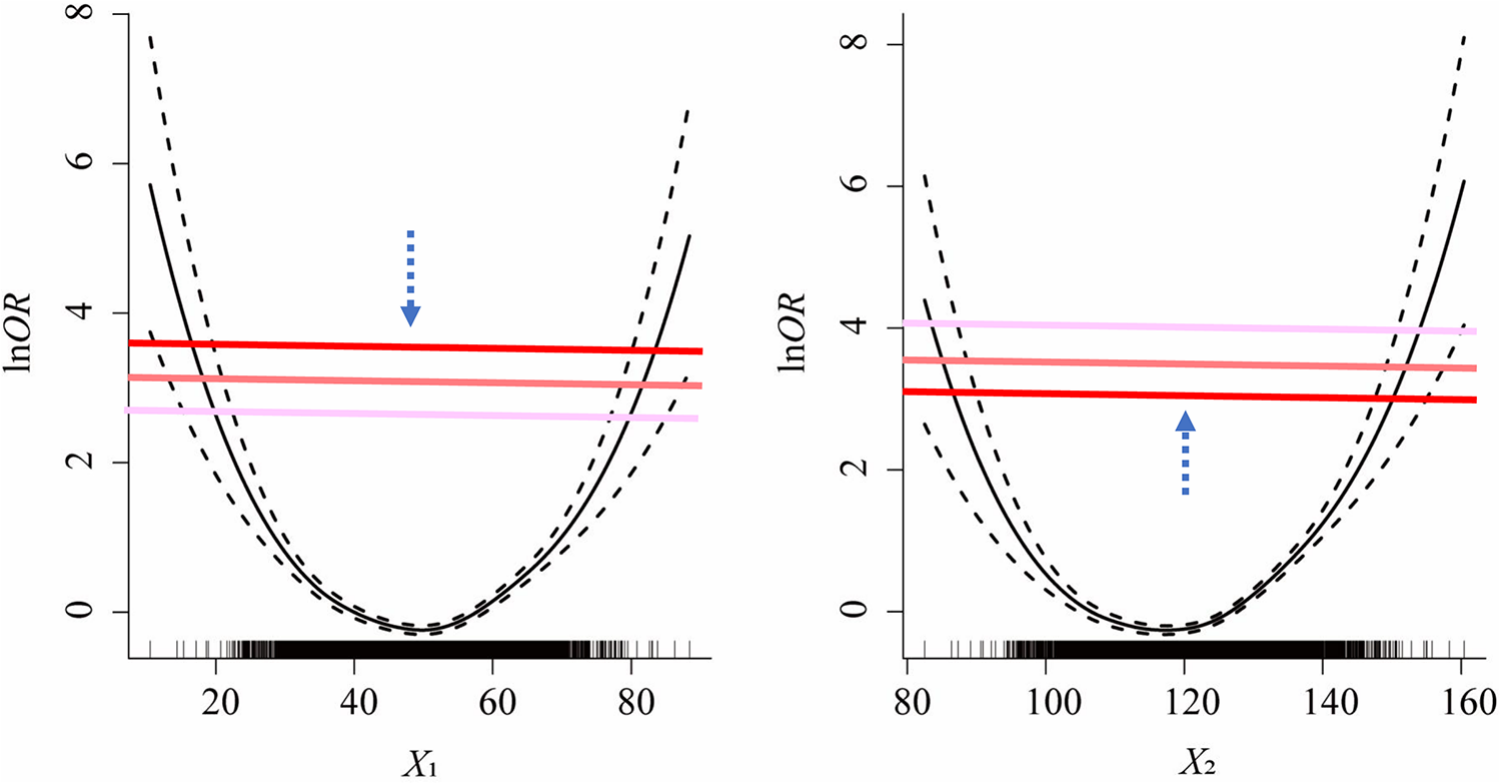
One of the scenarios of the scanning strategies for recursive gradient scanning method (i.e., scanning downward for *X*_1_ combined with upward for *X*_2_).

(1) If the curve depicted in the plot implies U-shaped associations between multiple independent variables and corresponding lnOR, we used the “two cut-points with maximum OR value method” for each variable to identify its optimal cut-points as their starting points for scanning, respectively.
(2) Find the percentile rankings of the estimated lnOR values for each independent variable, which are represented as *Q_k_*, *k *= 1, 2, …, 100. Subsequently, draw a horizontal line (known as “gradient”) parallel to the *x*-axis for each percentile between the 5th and the 95th percentile of the estimated lnOR. The *y*-value for each of these lines is set to *Q_k_*, *k *= 5, 6, …, 95. These lines intersect the fitted U-shaped curve at two points.
(3) Interpolation: The R-function spline interpolation technique is utilized to generate new data points as candidate cut-points, resulting in a smooth curve that maintains equal ln*OR* values across candidate cut-points (with a constraint for candidate cut-points that $|\ln OR_{1k}-\ln OR_{2k}|\;\leq \;0.01$).
(4) The recursive gradient scanning method: we set up a loop program to refine the boundary points and improve the discretization accuracy. Scanning starts from original cut-points of each variable and then moves up or down vertically in each gradient by the step of lnOR* *× 1/100. If the model fits increasingly well during the upward or downward scan, the model stops at the *P*_95_ of lnOR for the upward scan and at the *P*_5_ for the downward scan. If the model fits increasingly worse during the upward or downward scan, the current scan is suspended and the scan continues in the opposite direction. The number of independent variables determines the scanning method (e.g., if the number of variables is *k*, there are 2*^k^* scanning methods). Specifically, if *k *= 2, there are four scanning methods: (1) Scanning upward for *X*_1_ combined with downward for *X*_2_; (2) Scanning downward for *X*_1_ combined with upward for *X*_2_; (3) Scanning upward for both *X*_1_ and *X*_2_ simultaneously; (4) Scanning downward for both *X*_1_ and *X*_2_ simultaneously (illustrated in Figure 2).
(5) Select the best cut-points according to model fitness: We scan and calculate from the original cut-points in each program loop until the desired results are achieved. Then the goodness of fit index of the model under hyperparametric scenarios was obtained, such as AIC, Nagelkerke *R*^2^, and −2 log-likelihood. Select the respective variable cut-points corresponding to the best-fit model under each parameter combination as the final cut-points, place the discretized classified variables into the regression model, and rank the influencing factors according to the magnitude of OR values.

#### 2.5.5. Measures of predictive ability

The predictive performance of logistic regression models fitted with covariates discretized by different approaches was evaluated. The areas under the curve (AUC) constructed by receiver operating characteristic (ROC) analysis were calculated to compare different model's predictive capability.

#### 2.5.6. Implementation in R

The minimum *p*-value method with log-rank statistics was implemented using the R package “maxstat.” The freely available R package “SemiPar” was applied to fit logistic regression models with splines. The two-sided significance level for all tests was set at 0.05, and any *p-*values less than this threshold were deemed statistically significant. The R programming language, version 4.1.2 (R Foundation for Statistical Computing, http://www.R-project.org), was utilized for conducting the statistical analyses.

## 3. Results

### 3.1. Study population

**Figure 3:**
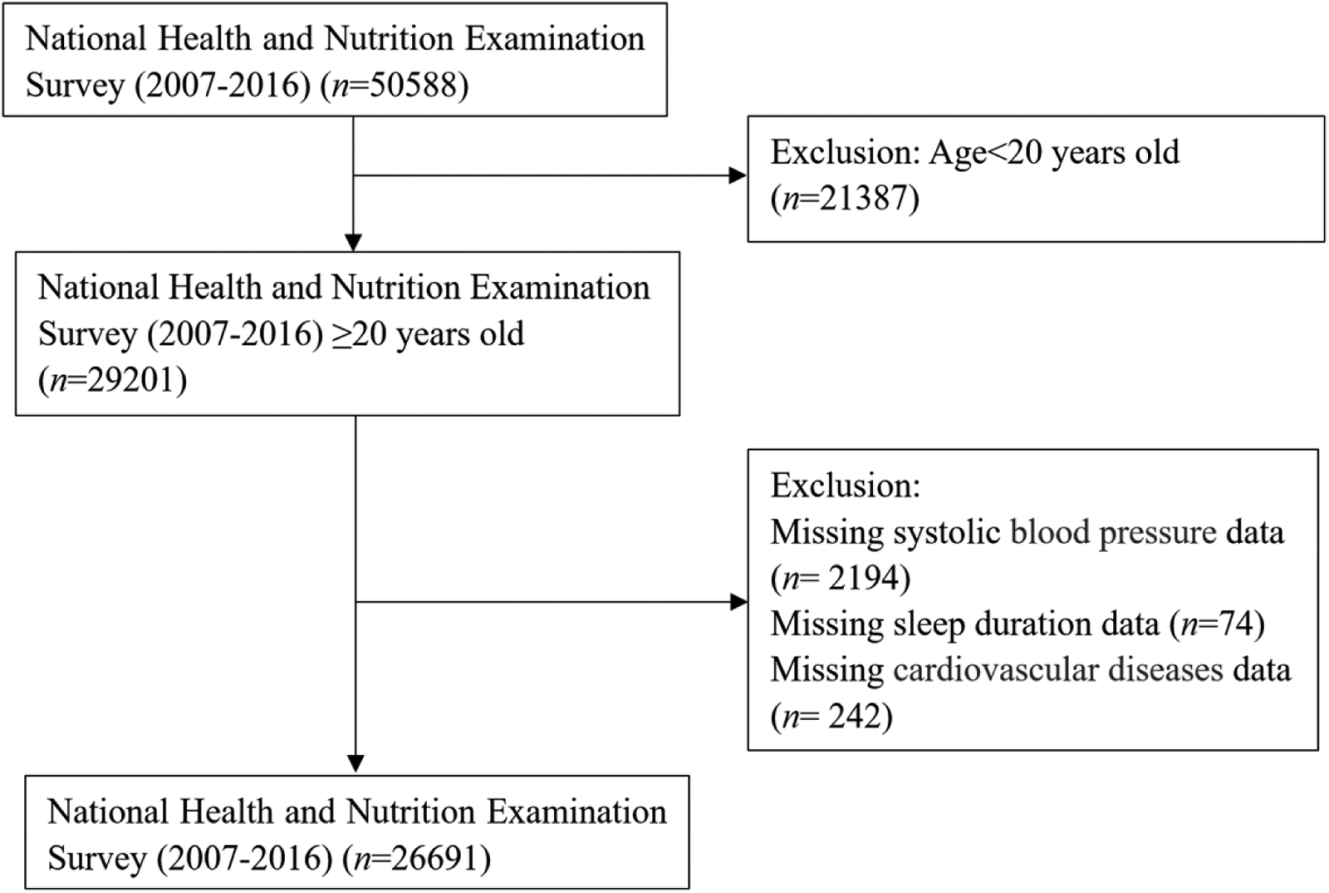
Flow chart for population selection. This figure represents the sample selection for the analysis of sleep duration as well as SBP with CVDs.

This study included a total of 26,691 participants from NHANES 2007–2016, with an average age of 49.4 years and 51.3% being female. Subjects younger than 20 years of age (*n *= 21,387) and those having missing data on SBP (*n *= 2,194), sleep duration (*n *= 74), and CVDs (*n *= 242) were excluded. Thus, 26,691 participants were included in the final list (Figure 3).

### 3.2. Characteristics of participants

The characteristics of study participants are presented in Table 1. Among all participants, 10.44% (2,786/26,991) reported having CVDs (826 CHF, 1,039 CHD, 636 angina pectoris, 1,062 heart attack, and 990 stroke). Compared with those without CVDs, participants with CVDs were more likely to be older, lacked physical activities, had higher levels of sedentary time, had higher prevalence of comorbidities, had more treatment, and had low income (all *p* < 0.0001). In addition, participants with CVDs had significantly higher SBP, fasting blood glucose, triglyceride, CRP and body mass index (BMI) (all *p* < 0.0001), indicating poor cardiometabolic risk profiles.

**Table 1:** The characteristics of participants in the NHANES 2007–2016 according to CVDs.

Participants’ characteristics	Total (*n *= 26,691)	CVDs		*p-*value
		No, *n *= 23,905	Yes, *n *= 2,786	
Age, years, mean (SD)	49.43 (17.69)	47.49 (17.15)	66.07 (12.81)	<0.0001
Sex, *n* (%)				<0.0001
Male	12,987 (48.66)	11,427 (47.80)	1,560 (55.99)	
Female	13,704 (51.34)	12,478 (52.20)	1,226 (44.01)	
Ethnicity, *n* (%)				<0.0001
Mexican American	4,081 (15.29)	3,818 (15.97)	263 (9.44)	
Other Hispanic	2,851 (10.68)	2,612 (10.93)	239 (8.58)	
Non-Hispanic White	11,173 (41.86)	9,705 (40.60)	1,468 (52.69)	
Non-Hispanic Black	5,670 (21.24)	5,041 (21.09)	629 (22.58)	
Other race	2,916 (10.93)	2,729 (11.41)	187 (6.71)	
Marital status, *n* (%)				<0.0001
Unmarried	7,080 (26.53)	6,743 (28.22)	337 (12.10)	
Married or Living with partner	13,668 (51.21)	12,272 (51.37)	1,396 (50.10)	
Divorced or Separated	5,943 (22.26)	4,876 (20.41)	1,053 (37.80)	
Education, *n* (%)				<0.0001
<9th grade	2,885 (10.82)	2,437 (10.20)	448 (16.11)	
9–11th grade	3,898 (14.62)	3,391 (14.20)	507 (18.23)	
High school	6,032 (22.62)	5,333 (22.33)	699 (25.13)	
Some college	7,724 (28.97)	7,021 (29.40)	703 (25.28)	
≥College graduate	6,127 (22.97)	5,703 (23.87)	424 (15.25)	
Income group, *n* (%)				<0.0001
Lowest, poverty income ratio ≤1.30	7,868 (29.48)	6,875 (28.76)	993 (35.64)	
Middle, poverty income ratio 1.31–1.85	3,076 (11.52)	2,701 (11.30)	375 (13.46)	
Highest, poverty income ratio ≥1.86	15,747 (59.00)	14,329 (59.94)	1,418 (50.90)	
Smoking, *n* (%)				<0.0001
Current	5,500 (20.62)	4,891 (20.48)	609 (21.87)	
Ever	6,351 (23.81)	5,281 (22.11)	1,070 (38.42)	
Never	14,820 (55.57)	13,714 (57.41)	1,106 (39.71)	
Alcohol, *n* (%)				<0.0001
Moderate alcohol	11,293 (46.14)	11,472 (47.99)	846 (30.37)	
Excessive alcohol	3,413 (13.94)	3,340 (13.97)	384 (13.79)	
Ever	6,117 (24.99)	5,546 (23.20)	1,120 (40.19)	
Never	3,653 (14.93)	3,547 (14.84)	436 (15.65)	
Sitting, min/day, Mean (SD)	356.44 (203.12)	352.25 (201.89)	392.47 (210.06)	<0.0001
Physical activity, *n* (%)				<0.0001
MET < 600 min/week	15,588 (58.40)	13,602 (56.90)	1,986 (71.30)	
MET ≥ 600 min/week	11,103 (41.60)	10,303 (43.10)	800 (28.70)	
Sleep duration, hours, median (IQR)	7 (6–8)	7 (6–8)	7 (6–8)	0.0483
Systolic blood pressure, mmHg, mean (SD)	123.88 (18.47)	123.04 (17.94)	131.08 (21.19)	<0.0001
Fasting blood glucose, mmol/L, mean (SD)	5.74 (2.24)	5.64 (2.14)	6.57 (2.86)	<0.0001
High-density lipoprotein, mmol/L, mean (SD)	1.36 (0.42)	1.38 (0.42)	1.29 (0.41)	<0.0001
Triglyceride, mmol/L, mean (SD)	1.76 (1.51)	1.74 (1.51)	1.89 (1.45)	<0.0001
BMI, kg/m^2^, mean (SD)	29.10 (6.90)	28.95 (6.84)	30.42 (7.32)	<0.0001
CRP, mg/dl, median (IQR)	0.20 (0.08–0.46)	0.19 (0.07–0.44)	0.27 (0.11–0.61)	0.0001
Hypertension, *n* (%)				<0.0001
No	14,495 (54.26)	13,898 (58.14)	583 (20.91)	
Yes	12,196 (45.74)	10,006 (41.86)	2,203 (79.09)	
Diabetes mellitus, *n* (%)				<0.0001
No	23,276 (87.20)	21,419 (89.60)	1,855 (66.57)	
Yes	3,415 (12.80)	2,486 (10.40)	931 (33.43)	
Hyperlipidemia, *n* (%)				<0.0001
No	17,886 (62.21)	15,658 (65.50)	1,016 (36.49)	
Yes	8,805 (37.79)	8,247 (34.50)	1,770 (63.51)	
Antihypertensive treatment, *n* (%) Association between antihypertensive treatment				<0.0001
No	19,368 (72.53)	18,426 (77.08)	934 (33.52)	
Yes	7,323 (27.47)	5,479 (22.92)	1,852 (66.48)	
Glucose-lowering treatment, *n* (%)				<0.0001
No	23,567 (88.30)	21,646 (90.55)	1,921 (68.95)	
Yes	3,124 (11.70)	2,259 (9.45)	865 (31.05)	
Lipid-lowering treatment, *n* (%)				<0.0001
No	21,671 (81.19)	20,330 (85.04)	1,341 (48.13)	
Yes	5,020 (18.81)	3,575 (14.96)	1,445 (51.87)	

IQR, interquartile range.

### 3.3. The analyses of U-shaped relationship

**Figure 4:**
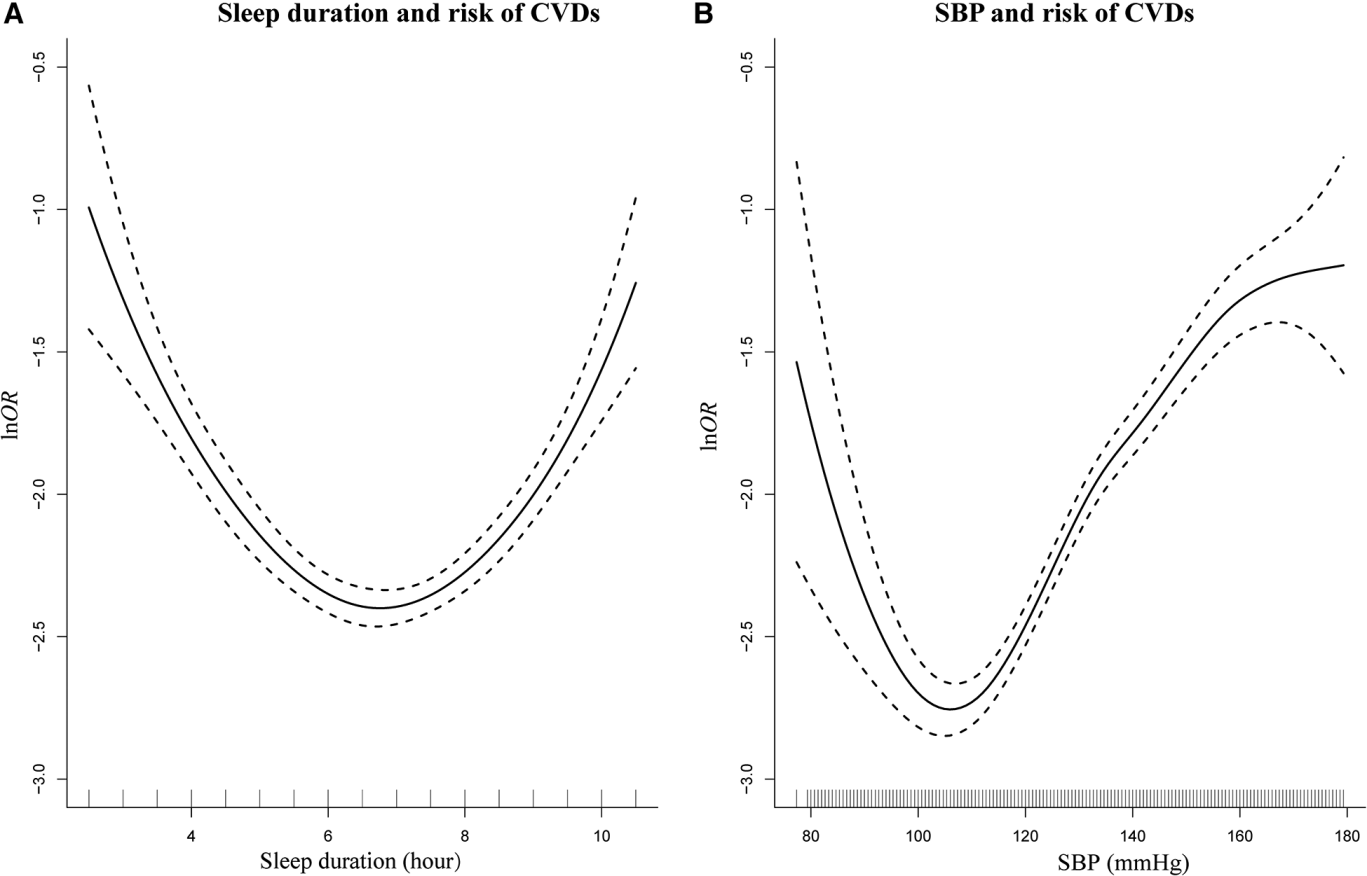
Smoothing plot for sleep duration and SBP with risks of CVDs by semiparametric regression analysis. U-shaped relationships were shown between sleep duration (**A**), SBP (**B**), and risks of CVDs [The solid line indicates the point estimation for ln odds ratios (lnOR) of CVDs, and the dotted lines represent 95% confidence intervals (CIs)].

As depicted in Figure 4, the results of the semiparametric regression analysis found a U-shaped association between sleep duration, SBP, and risks of CVDs. This U-shaped relationship suggested that individuals who sleep for intermediate duration and had healthier SBP levels were at a lower risk for CVDs. To better illustrate the relationships between all continuous variables and CVDs, we plotted the curve in Supplementary Figure S1.

### 3.4. Relationship between sleep duration, SBP, and CVDs by univariate logistic regression

The performance of logistic regression models for various estimated cut-points is illustrated in Table 2. The original method used to identify cut-points in the semiparametric regression analysis may not have been accurate. A new method, the recursive gradient scanning method, was used and significantly improved the model fitting effect (the new model had larger value in Nagelkerke *R*^2^ and smaller values in AIC and −2Loglikelihood). Our findings suggested that categorizing individuals into high-risk and low-risk groups based on the optimal cut-points of the U-shaped curve may offer a more precise depiction of the relationships between sleep duration and the risk of CVDs, as well as between SBP and the risk of CVDs.

**Table 2:** Performance of different estimated cut-points in logistic regression.

Cut-points	SBP*_L_* (mmHg)	SBP*_U_* (mmHg)	Sleep*_L_* (h)	Sleep*_U_* (h)	OR*s*_BP_ (95% CI)	OR_Sleep_ (95% CI)	AIC	*R* ^2^	−2 log-likelihood
Original cut-points	104	110	4.75	9.50	1.19 (1.10–1.30)	1.58 (1.44–1.74)	17,043	0.021	13,636.40
Optimal cut-points	95	120	6.50	8.00	1.59 (1.24–2.06)	1.35 (1.15–1.58)	16,962	0.027	13,579.28

*L* represents the lower cut-point*,* and *U* represents the upper cut-point.

Finally, we chose 6.5 and 8.0 h as optimal cut-points for sleep duration and chose 95 and 120 mmHg as optimal cut-points for SBP. Short and long sleep duration [OR* *= 1.35, 95% confidence interval (CI) = 1.15–1.58] was associated with a higher risk of CVDs (*p *< 0.0001). Participants with healthier SBP levels were at a lower risk for CVDs (OR* *= 1.59, 95% CI* *= 1.24–2.06).

### 3.5. Logistic regression model results after adjusting for covariates

As shown in Table 3, after adjustment for other risk factors, the OR for those with SBP greater than 120 or less than 95 mmHg was found to be 1.17 times greater than for those with SBP between 95 and 120 mmHg (OR* *= 1.17, 95% CI* *= 1.01–1.36; *p *= 0.0375). Similarly, individuals who slept more than 8.0 h per day or less than 6.5 h per day also had a higher risk for CVDs than those who slept between 6.5 and 8.0 h (OR* *= 1.20, 95% CI* *= 1.04–1.40; *p *= 0.0138).

**Table 3:** Multivariable logistic regression for the final model.

Variable	OR (95% CI)	Wald *χ*^2^	*p-*value
Sex	0.67 (0.56–0.79)	22.48	<0.0001
Ethnicity			
Mexican American (ref)			
Other Hispanic	1.12 (0.80–1.57)	0.4302	0.5119
Non-Hispanic White	1.68 (1.32–2.14)	17.79	<0.0001
Non-Hispanic Black	1.56 (1.18–2.07)	9.66	0.0019
Other race	1.40 (0.86–2.22)	1.97	0.1608
Income group			
Lowest, poverty income ratio ≤1.30 (ref)			
Middle, poverty income ratio 1.31–1.85	0.83 (0.65–1.06)	2.15	0.1430
Highest, poverty income ratio ≥1.86	0.71 (0.60–0.85)	14.88	0.0001
Smoking			
Never (ref)			
Ever	2.01 (1.61–2.49)	39.38	<0.0001
Current	1.38 (1.16–1.65)	12.98	0.0003
Alcohol			
Never (ref)			
Ever	0.80 (0.62–1.03)	3.12	0.0775
Moderate alcohol	1.06 (0.79–1.44)	0.15	0.6958
Excessive alcohol	1.19 (0.94–1.51)	2.13	0.1445
Physical activity			
MET < 600 min/week (ref)			
MET ≥ 600 min/week	0.77 (0.65–0.90)	10.67	0.0011
Sleep duration			
6.5–8.0 h per day (ref)			
<6.5 or >8.5 h per day	1.20 (1.04–1.40)	6.06	0.0138
SBP			
95–120 mmHg (ref)			
<95 or >120 mmHg	1.17 (1.01–1.36)	4.33	0.0375
Fasting blood glucose^a^, mmol/L	1.06 (1.03–1.09)	16.10	<0.0001
High-density lipoprotein^a^, mmol/L	0.60 (0.48–0.74)	22.23	<0.0001
BMI^#^, kg/m^2^	1.04 (1.02–1.05)	35.83	<0.0001

^a^ Variables were treated as continuous variable form.

### 3.6. Predictive ability and goodness-of-fit among different methods

The present study evaluated the predictive capacity of traditional discretization methods and compared it with an optimal model using ROC curve analysis. The results showed that the recursive gradient scanning method has a higher AUC value of 0.8344, indicating a better predictive capacity than traditional discretization methods. Moreover, the adjusted *R*^2^ value, which measured how well the model fits the data, was calculated and found to be 0.27812 for the recursive gradient scanning method, higher than the other traditional discretization methods, indicating a better fit of the model to the data. In addition, the goodness-of-fit index AIC was evaluated for all the methods. It was found that the recursive gradient scanning method had the lowest AIC value (AIC* *= 5,519.389), indicating that it provided an ideal compromise between model complexity and goodness of fit (Table 4).

**Table 4:** The predictive capacity and goodness-of-fit among different methods.

Method	AIC	AUC	Adjusted *R*^2^
The recursive gradient scanning method	5,519.389^(1)^	0.8344^(1)^	0.27812^(1)^
Minimum *p*-value	5,671.354^(2)^	0.8337^(4)^	0.27666^(4)^
Q1–Q3	5,778.388^(3)^	0.8342^(2)^	0.27795^(3)^
Median	6,658.365^(4)^	0.8341^(3)^	0.27809^(2)^

(1)–(4) means to rank according to the priority of the parameters for the model, and (1) means the highest priority.

### 3.7. Association patterns when covariates included

The following charts illustrated that additional adjustments for other covariates did not change the majority of our results. Furthermore, the associations and U-shaped trends between sleep duration, SBP, and CVD remained similar to our main results (Supplementary Figure S2).

## 4. Discussion

In this nationally representative survey of American adults, among 26,691 participants, 10.44% (sample *n *= 2,786) of the total reported having CVDs. This study suggested that a U-shaped relationship between sleep duration, SBP, and risk of CVDs, where both extremes of SBP and sleep duration are associated with an elevated risk of CVDs. Consequently, these results provide valuable insight into the potential impact of sleep duration and blood pressure on cardiovascular health.

In medical research, non-monotonic U-shaped dose–response relationships are increasingly common, and predictive models often involve multiple non-monotonic independent variables. If such kinds of explanatory variables are considered directly as candidate independents with a form of continuous variables in the regression model, their non-monotonic features may probably make themselves be eliminated in the followed selection, e.g., a stepwise selection. Therefore, the corresponding factors cannot be mentioned in the design of interventions. This study suggests to discretize factors according to their association with prognosis and to manifest their importance in assessing prognosis. Our previous pioneering research has garnered citations from experts in the field, highlighting the significance of our contributions. These citations from esteemed colleagues underscore the relevance and impact of our work, positioning it at the forefront of scholarly discourse. This recognition encourages us to continue our pursuit of advancing knowledge and making meaningful contributions to the field.

Unfortunately, there is a limited amount of research on discretization methods for multiple continuous variables, particularly in cases where there are U-shaped relationships between outcomes and explanatory variables. Choosing the appropriate discretization method is essential to obtain accurate predictions from statistical models. Therefore, it is critical to develop effective strategies for discretizing multiple continuous variables to ensure that these models are reliable and can be used to guide medical research and clinical decision-making. In this regard, it is essential to compare the predictive capacity of traditional discretization methods with optimal models. The results of our research showed that the recursive gradient scanning method had a higher AUC and adjusted *R*^2^ than other traditional discretization methods (including median, minimum *p*-value, and *Q*_1_–*Q*_3_), indicating its superior predictive capacity. Moreover, the goodness-of-fit index AIC remained the minimum for the recursive gradient scanning method in all the methods, further highlighting its efficacy in accurately predicting the outcomes of the statistical models. Overall, the results suggested that the recursive gradient scanning method was a promising approach for discretizing multiple non-monotonic independent variables, with potential applications in various fields. Further study is needed to explore the method's full potential and to compare it with other emerging discretization techniques, such as Bayesian classification (36, 37) and decision trees (38, 39).

Our study showed that having high or low SBP levels was associated with a 17% higher risk of CVDs compared to those with SBP within 95–120 mmHg intervals. Our study reinforces the importance of maintaining stable BP levels in managing CVDs. The concept of a U-shaped association between targeted SBP and the risk of morbidity and mortality has been long suggested (40). This hypothesis is based on the assumption of an SBP threshold for autoregulation of organ blood flow, and the potential role of BP as a compensatory mechanism for preserving organ function (41). The observed link between lower SBP and increased risk of CVDs supports the previous concerns about the intensity of antihypertensive treatment in older adults (42). Notably, low blood pressure can not only be harmful in itself but can also indicate poor health status (43). Even in physically fit individuals, low SBP was found to be associated with CVDs (44). However, the currently prevailing paradigm of “the lower, the better” in hypertension management has been challenged by recent randomized clinical trials (RCTs). The ACCORD trial involving diabetic patients (45) revealed that intensive blood pressure lowering (to 120 mmHg SBP) did not result in a decreased risk of cardiovascular outcomes compared to the standard therapy group (to 140 mmHg SBP). In contrast, the link between higher SBP and CVDs has been consistent in epidemiological studies. According to published studies, hypertension was found to be linked with a greater proportion of CVDs when compared to other common risk factors like smoking, obesity, hypercholesterolemia, and diabetes (46, 47). Taking into account the overall evidence, adopting a less aggressive treatment approach may be the optimal approach to manage hypertension (48). Our study expands upon these findings by showing that maintaining a stable SBP level is crucial in reducing the risk of CVDs.

Similarly, individuals who slept for more than 8 h or less than 6.5 h had a higher risk for CVDs than those who slept for 6.5–8 h. The OR for this group was 1.20, indicating that individuals who sleep beyond the normal range have a 20% higher risk of CVDs than those who sleep for the recommended duration (6.5–8.0 h). This finding supported the notion that maintaining optimal sleep durations were critical in reducing the risk of CVDs. Short sleep duration has been consistently associated with increased risks of CVDs in observational studies (6, 49, 50). The pathophysiological mechanisms underlying this association involve abnormalities in the sympathetic nervous system, acceleration of arterial stiffening and atherosclerosis, increased inflammation, and cardiac dysfunction (5, 51, 52). Recent studies have suggested that extended sleep duration could improve cardiovascular health, particularly in college students or prehypertension participants who are often sleep-deprived (53, 54). Therefore, increasing sleep duration among individuals with short sleep may be a promising strategy to reduce the risk of CVDs. On the contrary, some studies have proposed that a longer duration of sleep is linked to a higher risk of developing cardiovascular disease and cardiometabolic disease (55, 56). Physiological changes that could happen include elevated blood pressure (57), impaired glucose metabolism (58), and a rise in cortisol levels (59). Furthermore, there was evidence indicating that a longer duration of sleep is linked to an increase in carotid intima-media thickness (60). Therefore, it may be clinically recommended to advise individuals with prolonged sleep duration to reduce their sleep time.

The current study has several strengths, including the utilization of the recursive gradient scanning method to discretize multiple non-monotonic independent variables. This method provides valuable insights into the relationship between sleep duration, SBP, and the risk of CVDs. Another strength is that we screened multiple influencing factors and then sorted them according to the size of the OR value, which corresponds to the risk level of each influencing factor. It will be helpful to indicate priorities for formulating intervention measures. People at a greater risk of CVDs may benefit significantly from public health campaigns promoting good sleep hygiene in the future.

To properly evaluate the outcomes of this study, it is crucial to recognize several limitations. First of all, the cross-sectional design of NHANES imposes restrictions on establishing causality or accurately determining the direction of the relationship. Thus, it is important to acknowledge the limitations of observational data and the potential for reverse causation when drawing conclusions about causality. Whenever possible, randomized trials and prospective studies can provide more robust evidence for establishing causal relationships. Second, our understanding of the associations may have been underestimated due to the absence of information about the changes over time in sleep duration and SBP, as we only had baseline assessments of these markers. The approach of utilizing mean values from short-term repeated BP measurements to assess CVD risk fails to account for BP variability and inadequately addresses masked hypertension and white coat hypertension. Instead, 24-h ambulatory blood pressure monitoring (ABPM) provides continuous readings, capturing natural fluctuations, nighttime levels, and patterns such as non-dipping. Consideration of a combined approach involving clinic-based and periodic ABPM may yield a more comprehensive evaluation of BP dynamics and CVD risk in certain cases. Third, the study relied solely on self-reported sleep duration data, which could lead to measurement errors and potentially impact the precision of the findings. Future research could benefit from using objective measures of sleep duration, such as polysomnography or actigraphy, to improve data reliability. Furthermore, despite careful adjustment for many potential confounding factors to ensure the validity of the key findings, residual confounding may still exist due to unmeasured risk factors. Therefore, additional research is required to replicate these associations and investigate the mechanisms underlying the results.

## 5. Conclusion

U-shaped relationships were identified between sleep duration, BP, and risk of CVDs. Both shorter and longer sleep duration/higher and lower SBP are significant predictors of CVDs in large population studies. One should consider duration of sleep and blood pressure control as additional behavioral risk factors that are heavily influenced by environmental factors and can potentially be modified through education, counseling, and public health interventions.

## Data Availability

Publicly available datasets were analyzed in this study. These data can be found here: Centers for Disease Control and Prevention (CDC), National Center for Health Statistics (NCHS), National Health and Nutrition Examination Survey (NHANES), https://wwwn.cdc.gov/nchs/nhanes/default.aspx.
