# Supplementary figures and images for: Examining the U-shaped relationship of sleep duration and systolic blood pressure with risk of cardiovascular events using a novel recursive gradient scanning model
Source: Front Cardiovasc Med. 2023 Sep 14;10:1210171. doi: 10.3389/fcvm.2023.1210171 (PMC10543086; doi:10.3389/fcvm.2023.1210171)

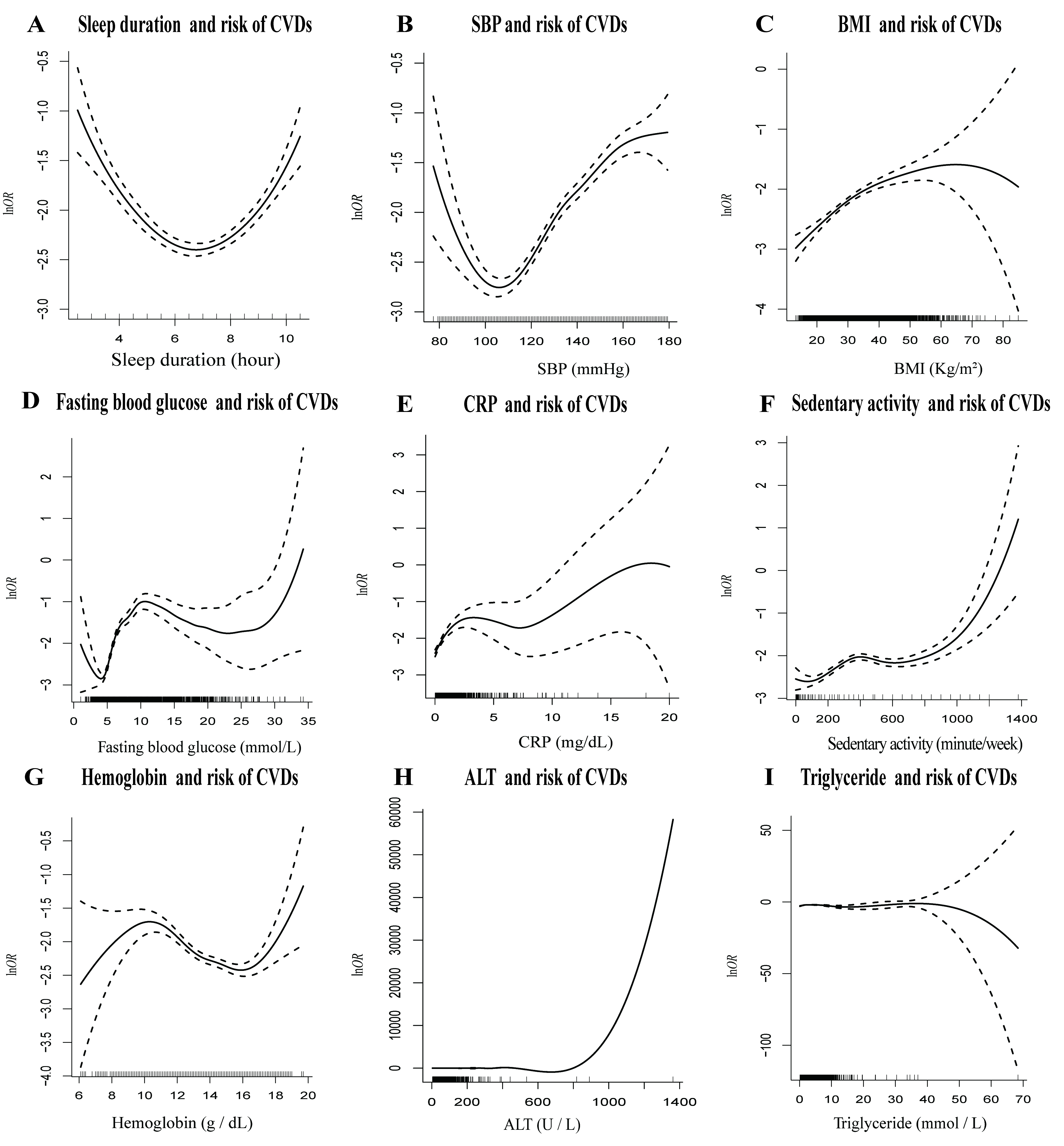

Supplement: Supplementary file 1 [file Image1.tif]

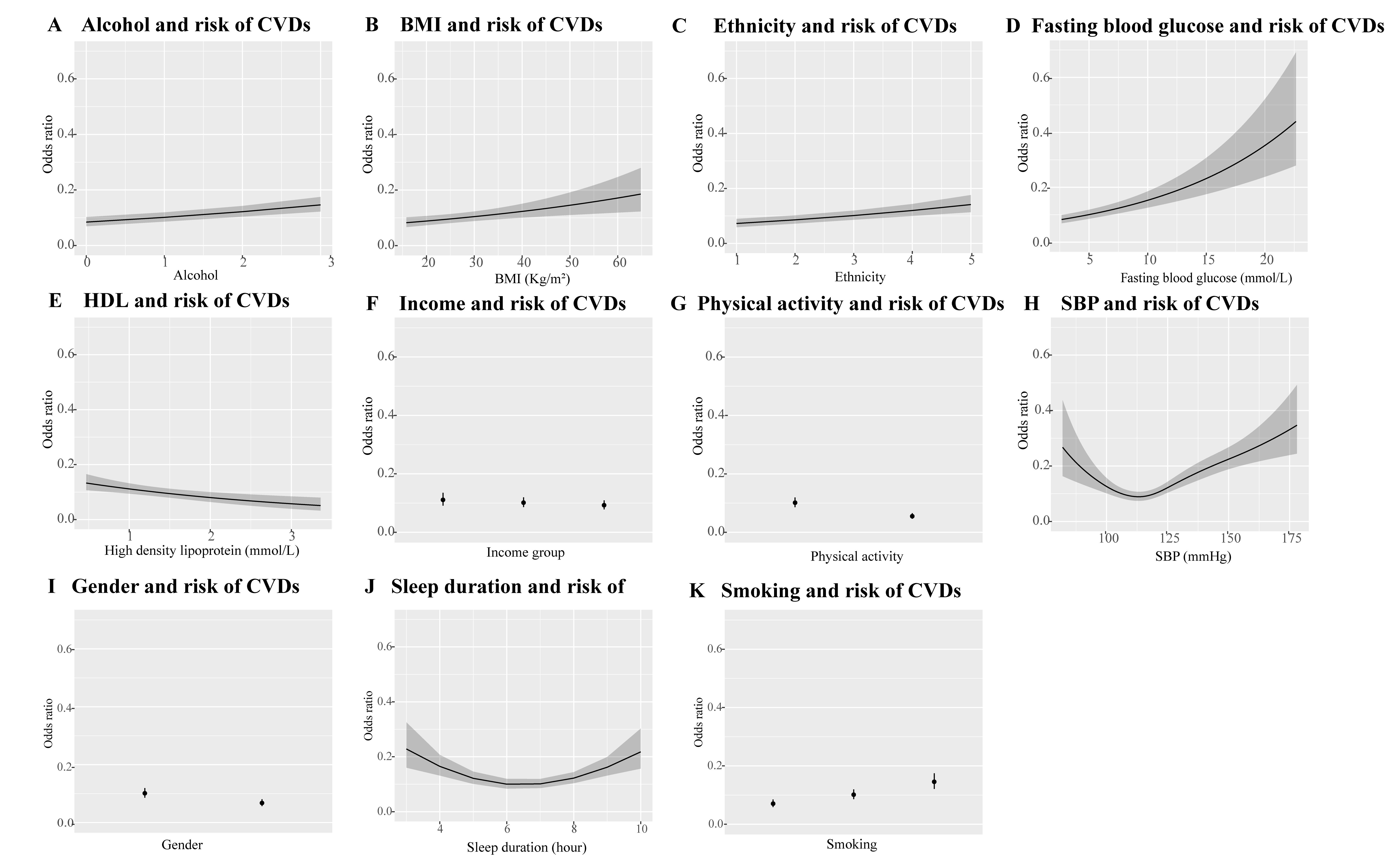

Supplement: Supplementary file 2 [file Image2.tif]
